# Supplementary material for: Targeting Host Glycolysis as a Strategy for Antimalarial Development
Source: Front Cell Infect Microbiol. 2021 Sep 16;11:730413. doi: 10.3389/fcimb.2021.730413 (PMC8482815; doi:10.3389/fcimb.2021.730413)
Supplement: Supplementary file 1 [file DataSheet_1.pdf]

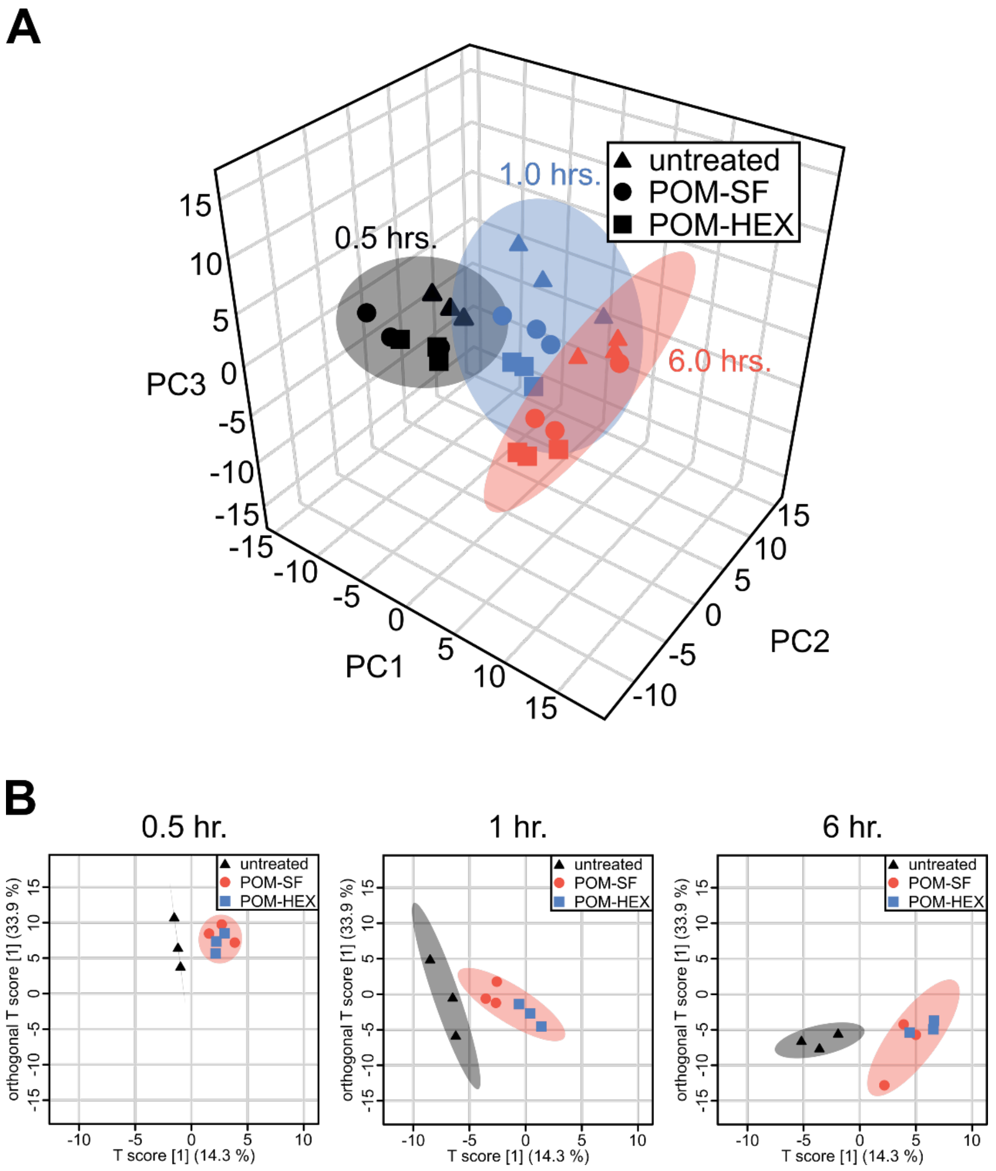

Supplemental Figure 1: Consistent metabolic signature of glycolytic inhibition. (A) Principle components analysis (PCA) of erythrocytes with and without treatment with enolase inhibitors (POM-SF, 2.4 μM, and POM-HEX, 7 μM) demonstrates distinct separation of samples with respect to time and condition, illustrated by 95% confidence intervals of a minimum volume enclosing ellipsoid for each time point, teal = 0.5 hr., pink = 1 hr., blue = 6 hr. (B) Within each time point there is clear separation of treated samples (circles and squares) from untreated controls (plus symbol) as more clearly illustrated using orthogonal projections to latent structures discriminant analysis (OPLS-DA).

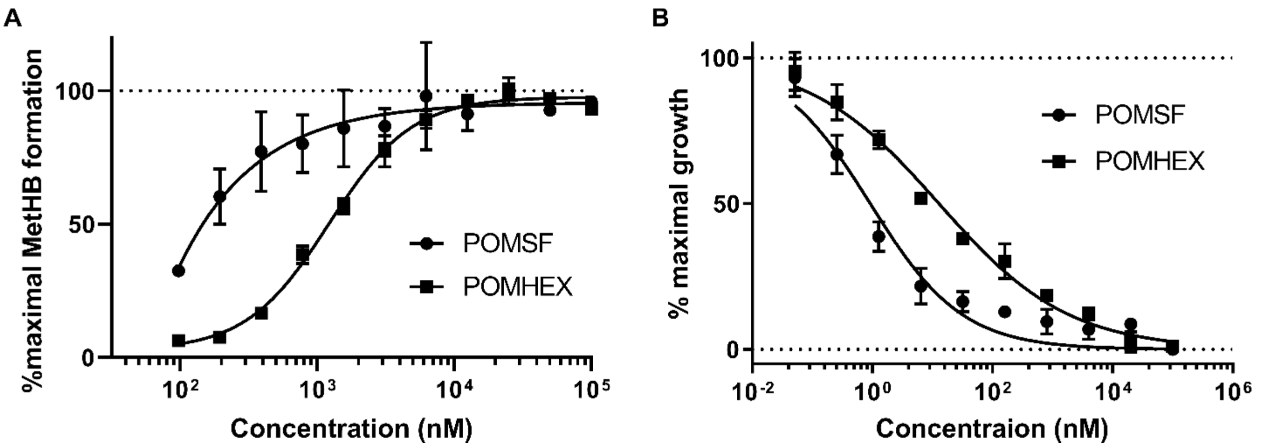

Supplemental Figure 2: Methemoglobin formation and parasite growth inhibition without pyruvate supplementation. Dose response curves are representative of three independent experimental replicates. Non-linear regression was performed using GraphPad Prism.

Additional data file

Supplemental Table 1. Untargeted LC/MS of POM-SF and POM-HEX treated erythrocytes with two-way repeated measures analysis of variance (ANOVA).

Sample	Phenotype	Time	PC1	PC2	PC3	Score (t1)	OrthoScore (to1)
S1T0.5	Untreated	0.5	-11.187	4.0977	-0.50702	-1.5272	10.808
S1T1	Untreated	1	-5.2149	8.6656	4.4176	-8.5524	5.0246
S1T6	Untreated	6	6.3029	6.6861	0.24052	-5.2479	-6.5711
S2T0.5	Untreated	0.5	-3.6239	-1.1955	2.6787	-0.97074	3.8803
S2T1	Untreated	1	5.766	5.0932	3.0898	-6.2673	-5.7055
S2T6	Untreated	6	7.4986	4.2406	0.9167	-3.6426	-7.6177
S3T0.5	Untreated	0.5	-6.4935	0.68517	1.5603	-1.1952	6.5305
S3T1	Untreated	1	0.48451	3.9959	5.1811	-6.5399	-0.34779
S3T6	Untreated	6	5.4986	1.8652	0.79194	-1.98	-5.5483
S4T0.5	POMSF	0.5	-9.1342	-6.7371	3.8178	2.6948	9.6091
S4T1	POMSF	1	-1.5747	1.2281	2.2968	-2.6176	1.6842
S4T6	POMSF	6	13.608	-5.9649	7.9595	2.1306	-12.902
S5T0.5	POMSF	0.5	-6.6397	-6.8714	2.5662	3.8568	7.0615
S5T1	POMSF	1	1.3937	1.8705	1.7246	-2.6998	-1.2993
S5T6	POMSF	6	5.8559	-4.7354	-1.2115	4.9324	-5.798
S6T0.5	POMSF	0.5	-8.6315	1.7468	-4.032	1.5729	8.3196
S6T1	POMSF	1	0.43739	5.597	-2.188	-3.5977	-0.70088
S6T6	POMSF	6	3.7712	1.4584	-7.3746	3.849	-4.3323
S7T0.5	POMHEX	0.5	-8.2408	-3.9992	0.16622	2.9608	8.3721
S7T1	POMHEX	1	3.209	-4.12	2.6662	0.35493	-2.7783
S7T6	POMHEX	6	3.5165	-3.6213	-6.4734	6.5437	-3.8174
S8T0.5	POMHEX	0.5	-5.4074	-2.5732	-0.23804	2.1494	5.5285
S8T1	POMHEX	1	1.5453	0.018894	-0.78583	-0.63661	-1.4763
S8T6	POMHEX	6	4.8119	-4.0887	-5.9501	6.4988	-5.0309
S9T0.5	POMHEX	0.5	-7.4582	0.079372	-4.0643	2.2136	7.2059
S9T1	POMHEX	1	4.9242	-3.6691	0.76423	1.3414	-4.6182
S9T6	POMHEX	6	4.983	0.24724	-8.0134	4.3758	-5.4802

Supplemental Table 2. Principle components analysis (PCA) and orthogonal projections to latent structures discriminant analysis (OPLS-DA). Sample names reflect the sample number followed by the time the sample was collected in units of hours. PCA results from this table are plotted in Supplemental Figure 1A as a three-dimensional projection in two-dimensional space. OPLS-DA Results from this table are plotted in Supplemental Figure 1B for each time point comparing all samples

Metabolic pathway analysis								
	**Total**	**Expected**	**Hits**	**Raw p**	**-LOG(p)**	**Holm adj.**	**FDR**	**Impact**
Aminoacyl-tRNA biosynthesis	75	0.6855	10	2.25E-10	22.215	1.80E-08	1.80E-08	0.16902
Nitrogen metabolism	39	0.35646	8	6.06E-10	21.225	4.79E-08	2.42E-08	0.0083
Alanine, aspartate and glutamate metabolism	24	0.21936	5	1.49E-06	13.416	0.000116	3.98E-05	0.69421
Cyanoamino acid metabolism	16	0.14624	4	8.88E-06	11.632	0.000684	0.000178	0
Glycine, serine and threonine metabolism	48	0.43872	5	5.21E-05	9.8624	0.003959	0.000834	0.42039
Glutathione metabolism	38	0.34732	4	0.000315	8.0617	0.023655	0.004205	0.24838
Cysteine and methionine metabolism	56	0.51184	4	0.001408	6.5656	0.10419	0.01609	0.03581
Valine, leucine and isoleucine biosynthesis	27	0.24678	3	0.001683	6.3874	0.12283	0.016827	0.03498
D-Glutamine and D-glutamate metabolism	11	0.10054	2	0.004174	5.4789	0.30052	0.036556	0.13904
Arginine and proline metabolism	77	0.70378	4	0.00457	5.3883	0.32444	0.036556	0.03582
Phenylalanine metabolism	45	0.4113	3	0.007337	4.9149	0.51357	0.053358	0.11906
Glyoxylate and dicarboxylate metabolism	50	0.457	3	0.009838	4.6215	0.67883	0.065587	0.01316
Citrate cycle (TCA cycle)	20	0.1828	2	0.013719	4.289	0.93287	0.078392	0.15351
Taurine and hypotaurine metabolism	20	0.1828	2	0.013719	4.289	0.93287	0.078392	0.35252
Thiamine metabolism	24	0.21936	2	0.019493	3.9377	1	0.10396	0
Pantothenate and CoA biosynthesis	27	0.24678	2	0.024384	3.7138	1	0.11475	0
Phenylalanine, tyrosine and tryptophan biosynthesis	27	0.24678	2	0.024384	3.7138	1	0.11475	0.008
Methane metabolism	34	0.31076	2	0.037502	3.2834	1	0.16668	0.01751
Purine metabolism	92	0.84088	3	0.049189	3.0121	1	0.20182	0.00791
Butanoate metabolism	40	0.3656	2	0.050455	2.9867	1	0.20182	0.08516
Nicotinate and nicotinamide metabolism	44	0.40216	2	0.059869	2.8156	1	0.21771	0
Histidine metabolism	44	0.40216	2	0.059869	2.8156	1	0.21771	0.00051
Porphyrin and chlorophyll metabolism	104	0.95056	3	0.066468	2.711	1	0.22434	0
Primary bile acid biosynthesis	47	0.42958	2	0.067302	2.6986	1	0.22434	0.01644
Tyrosine metabolism	76	0.69464	2	0.15164	1.8862	1	0.47028	0.04724
Sulfur metabolism	18	0.16452	1	0.15284	1.8784	1	0.47028	0
Ether lipid metabolism	23	0.21022	1	0.19117	1.6546	1	0.56642	0
Sphingolipid metabolism	25	0.2285	1	0.20603	1.5797	1	0.58865	0
beta-Alanine metabolism	28	0.25592	1	0.22783	1.4791	1	0.60094	0
Glycolysis or Gluconeogenesis	31	0.28334	1	0.24907	1.39	1	0.60094	0.0953
Pentose phosphate pathway	32	0.29248	1	0.25602	1.3625	1	0.60094	0
Lysine biosynthesis	32	0.29248	1	0.25602	1.3625	1	0.60094	0

Vitamin B6 metabolism	32	0.29248	1	0.25602	1.3625	1	0.60094	0.01914
Pyruvate metabolism	32	0.29248	1	0.25602	1.3625	1	0.60094	0.18254
Terpenoid backbone biosynthesis	33	0.30162	1	0.26291	1.3359	1	0.60094	0
Ubiquinone and other terpenoid-quinone biosynthesis	36	0.32904	1	0.28322	1.2615	1	0.62939	0
Glycerophospholipid metabolism	39	0.35646	1	0.303	1.194	1	0.65153	0.01641
Valine, leucine and isoleucine degradation	40	0.3656	1	0.30948	1.1729	1	0.65153	0.02232
Folate biosynthesis	42	0.38388	1	0.32225	1.1324	1	0.66104	0.03372
Ascorbate and aldarate metabolism	45	0.4113	1	0.341	1.0759	1	0.682	0.01617
Lysine degradation	47	0.42958	1	0.35322	1.0407	1	0.68921	0
Pentose and glucuronate interconversions	53	0.48442	1	0.3886	0.9452	1	0.74019	0
Pyrimidine metabolism	60	0.5484	1	0.42754	0.8497	1	0.79543	0

Metabolite-protein interaction nodal analysis

Pathway	Total	Expected	Hits	P.Value	FDR			
Glutathione metabolism	46	1.26	37	4.84E-52	1.05E-49			
Alanine, aspartate and glutamate metabolism	32	0.875	25	2.11E-34	2.29E-32			
Pyruvate metabolism	41	1.12	19	7.61E-20	5.51E-18			
Metabolism of xenobiotics by cytochrome P450	71	1.94	19	1.37E-14	7.43E-13			
Cysteine and methionine metabolism	34	0.93	13	1.48E-12	6.42E-11			
Glycine, serine and threonine metabolism	33	0.903	12	2.28E-11	8.23E-10			
Arginine and proline metabolism	56	1.53	13	1.81E-09	5.61E-08			
Arachidonic acid metabolism	63	1.72	13	8.52E-09	2.31E-07			
Taurine and hypotaurine metabolism	9	0.246	6	2.93E-08	7.06E-07			
Glycolysis / Gluconeogenesis	65	1.78	12	1.23E-07	2.66E-06			
Phenylalanine, tyrosine and tryptophan biosynthesis	5	0.137	4	2.62E-06	5.16E-05			
Proximal tubule bicarbonate reclamation	7	0.191	4	1.75E-05	0.000317			
Citrate cycle (TCA cycle)	30	0.821	6	0.000129	0.00215			
Selenocompound metabolism	12	0.328	4	0.000223	0.00346			
Phenylalanine metabolism	17	0.465	4	0.000964	0.0139			
Valine, leucine and isoleucine degradation	44	1.2	5	0.00654	0.0887			
Tyrosine metabolism	33	0.903	4	0.0118	0.151			
Glyoxylate and dicarboxylate metabolism	19	0.52	3	0.0141	0.155			
One carbon pool by folate	19	0.52	3	0.0141	0.155			

Aminoacyl-tRNA biosynthesis	7	0.191	2	0.0142	0.155			
Sulfur relay system	8	0.219	2	0.0187	0.193			
Glycerophospholipid metabolism	82	2.24	6	0.0239	0.236			
Butanoate metabolism	25	0.684	3	0.0296	0.28			
beta-Alanine metabolism	26	0.711	3	0.0328	0.297			

Supplemental Table 3. Metabolic pathway analysis and metabolite-protein interaction nodal analysis.

Compound	Structure	Parasite EC_{50} (μM)	MetHb EC_{50} (μM)	S.I.	Parasite EC_{50} (μM)	MetHb EC_{50} (μM)	S.I.	S.I. Fold Change
		- pyruvate			+ pyruvate			
HEX	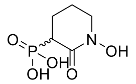	19 ± 1.0	500	26.3	18 ± 2.2	500	27.8	1.1
SDR22-2	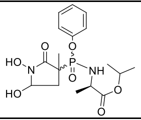	30 ± 1.1	500	16.7	55 ± 7.9	500	9.1	0.5
SDR4	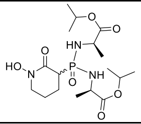	32 ± 2.4	500	15.6	73 ± 8.7	500	6.8	0.4
PKM J45	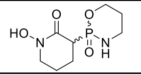	35 ± 3.1	500	14.3	94 ± 4.7	500	5.3	0.4
SDR6	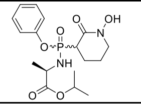	48 ± 2.8	500	10.4	60 ± 13	500	8.3	0.8
DeoxySF-2312	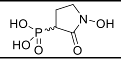	80 ± 2.0	500	6.3	30 ± 9.7	500	16.7	2.7
MethylSF-2312	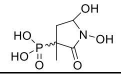	130 ± 3.5	500	3.8	26 ± 2.9	500	19.2	5.1
SF2312	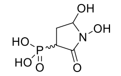	180 ± 9.3	500	2.8	27 ± 2.0	500	18.5	6.6
MethoxySF-2312	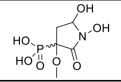	420 ± 64	500	1.2	91 ± 0.36	500	5.5	4.6
FluoroSF-2312	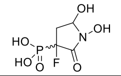	500	500	1.0	190 ± 24	500	2.6	2.6
J61	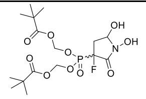	2.1 ± 0.06	1.8 ± 0.39	0.9	0.61 ± 0.11	5.2 ± 0.29	8.5	9.9
J42	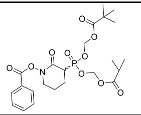	3.0 ± 0.25	1.7 ± 0.51	0.6	6.0 ± 4.0	3.0 ± 0.02	0.5	0.9
SDR23	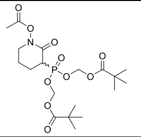	2.1 ± 0.25	1.3 ± 0.07	0.6	1.6 ± 0.11	2.5 ± 0.34	1.6	2.5
POM-HEX	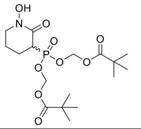	2.9 ± 0.64	1.2 ± 0.19	0.4	0.91 ± 0.15	1.4 ± 0.11	1.5	3.7
J52	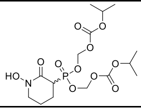	2.9 ± 0.40	0.77 ± 0.04	0.3	1.6 ± 4.0	0.49 ± 0.02	0.3	1.2
POM-SF	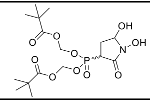	0.71 ± 0.04	0.1 ± 0.002	0.1	0.25 ± 0.01	0.48 ± 0.01	1.9	13.6

Supplemental Table 4. Table of parasite EC_{50}s and methemoglobin EC_{50}s for all tested compounds and their structures.

Cell line	POM-SF EC_{50} (nM)	POM-HEX EC_{50} (nM)
P. falciparum 3D7 (pan-sensitive)	185 ± 18	910 ± 148
P. falciparum K1 (chloroquine and sulfadoxine-pyrimethamine resistant)	361 ± 34	1112 ± 530
P. falciparum D10 (mefloquine resistant)	437 ± 83	1432 ± 540
P. falciparum IPC-5202 (chloroquine and artemisinin resistant)	321 ± 11	966 ± 442

Supplemental Table 5. Enolase inhibitors are active against multidrug-resistant *P. falciparum*. Half-maximal inhibitor concentrations (EC_{50}s) were determined for drug resistant malaria parasites and compared to the sensitive 3D7 strain. The respective EC_{50}s are calculated from each of the independent biological replicates using a non-linear regression of the log of the inhibitor concentration with data from each technical replicate normalized to maximal and minimal growth using the software package GraphPad Prism. Displayed are the means ± s.e.m calculated from three biological replicates.
